# Tissue Engineering and Cell-Based Therapies for Fractures and Bone Defects

**DOI:** 10.3389/fbioe.2018.00105

**Published:** 2018-07-31

**Authors:** Jose R. Perez, Dimitrios Kouroupis, Deborah J. Li, Thomas M. Best, Lee Kaplan, Diego Correa

**Affiliations:** ^1^Department of Orthopedics, UHealth Sports Medicine Institute, Miller School of Medicine, University of Miami, Miami, FL, United States; ^2^Diabetes Research Institute & Cell Transplant Center, Miller School of Medicine, University of Miami, Miami, FL, United States

**Keywords:** tissue engineering, stem cells, bone defects, fracture repair, biomaterials, mechanical stimulation, avascular necrosis, non-union

## Abstract

Bone fractures and segmental bone defects are a significant source of patient morbidity and place a staggering economic burden on the healthcare system. The annual cost of treating bone defects in the US has been estimated to be $5 billion, while enormous costs are spent on bone grafts for bone injuries, tumors, and other pathologies associated with defective fracture healing. Autologous bone grafts represent the gold standard for the treatment of bone defects. However, they are associated with variable clinical outcomes, postsurgical morbidity, especially at the donor site, and increased surgical costs. In an effort to circumvent these limitations, tissue engineering and cell-based therapies have been proposed as alternatives to induce and promote bone repair. This review focuses on the recent advances in bone tissue engineering (BTE), specifically looking at its role in treating delayed fracture healing (non-unions) and the resulting segmental bone defects. Herein we discuss: (1) the processes of endochondral and intramembranous bone formation; (2) the role of stem cells, looking specifically at mesenchymal (MSC), embryonic (ESC), and induced pluripotent (iPSC) stem cells as viable building blocks to engineer bone implants; (3) the biomaterials used to direct tissue growth, with a focus on ceramic, biodegradable polymers, and composite materials; (4) the growth factors and molecular signals used to induce differentiation of stem cells into the osteoblastic lineage, which ultimately leads to active bone formation; and (5) the mechanical stimulation protocols used to maintain the integrity of the bone repair and their role in successful cell engraftment. Finally, a couple clinical scenarios are presented (non-unions and avascular necrosis—AVN), to illustrate how novel cell-based therapy approaches can be used. A thorough understanding of tissue engineering and cell-based therapies may allow for better incorporation of these potential therapeutic approaches in bone defects allowing for proper bone repair and regeneration.

## Introduction

Fracture healing typically occurs uninterrupted during the first 6–8 weeks following an injury, although this process can be delayed by structural parameters such as the presence of thick cortices, which require more time to heal, as well as unfavorable mechanical and biological environments generated from excessive fracture site movement and/or gaps to general factors including aging, alcohol, tobacco, and steroid abuse and medical conditions such as infection, type 1 diabetes, anemia, and deficient nutrition (Kostenuik and Mirza, 2017). Occasionally, a combination of these unfavorable environments results in impaired fracture healing as damaged vascular supply and periosteum coincides with polytrauma or soft tissue damage (Gómez-Barrena et al., 2015). As a result, delayed union or even nonunion can occur following failed fracture healing after 4 or 6 months, respectively, ranging from 1.7% up to 18.5%. These fracture healing disruptions remain major orthopedic complications as they are not only a health care burden but also present significant challenges to the surgeon given the underlying biological difficulties re-routing bone structures toward adequate healing (Fong et al., 2013; Gómez-Barrena et al., 2015). This also raises the potential for increased rates of critical sized bone defects, which are more than 2 cm in length involving over half the circumference of the affected bone, resulting from failed spontaneous healing despite surgical intervention (Watanabe et al., 2016).

In an effort to treat these defects, different types of bone grafts have been used including autografts, allografts, and synthetic grafts. Allografts have several drawbacks including graft rejection and disease transmission, while some synthetic grafts show an increased susceptibility to wear and tear (Salgado et al., 2004). Autologous bone grafts, on the other hand, are considered the gold standard to treat bone defects due to their established osteoinductive and osteoconductive properties, obviating the histocompatibility issue. When compared to allografts, autografts result in shorter time to union (Flierl et al., 2013). Even though autografts constitute a popular graft option, they also have drawbacks such as donor site morbidity, muscle weakness, and surgical complications such as pain, hemorrhage, infection, and nerve injury at the donor site (Younger and Chapman, 1989). In addition to these complications, these grafts have a staggering impact on healthcare costs. An estimated 1.6 million bone grafts are used annually in the U.S. for degenerative diseases, injuries, tumors, and infections, accounting for approximately $244 billion, with trauma and fracture management representing almost 40% of those costs (O'Keefe and Mao, 2011).

Furthermore, after 10 years of incorporation, as high as 60% of grafts may fail to integrate leading to nonunions and late graft fractures (Soucacos et al., 2006). In an effort to find alternative therapies to treat bony defects and the complications associated with them, bone tissue engineering (BTE) has grown in popularity and is now being studied as a possible alternative in fracture management. This review discusses the process by which bone formation occurs and the role BTE may play by examining its different components including: (1) stem cells, (2) biomaterials, (3) growth factors, and (4) mechanical stimulation. Finally, we review the role of stem cell therapies in bone formation to treat non-unions and avascular necrosis of the femoral head, with and without core decompression.

## Bone formation and fracture healing

### Physiological bone formation

During development, skeletal and craniofacial structures form following two distinct processes, endochondral and intramembranous ossification, respectively, yet with some exceptions. Endochondral ossification is a process which leads to the formation of the axial and appendicular skeleton including the medial half of the clavicle and fragments of the scapula, through the formation of primary and secondary ossification centers. The cascade of endochondral bone formation begins with the differentiation of mesenchymal precursors into chondroblasts at the center of long bone condensations. The resulting chondrocytes in this avascular environment form the framework for subsequent bone growth by secreting a matrix consisting primarily of collagen II and proteoglycans. Chondrocytes stop to proliferate and differentiate into various intermediate phenotypes culminating in hypertrophic cells. These hypertrophic chondrocytes participate in the capillary invasion into the cartilage (White and Wallis, 2001), where osterix-expressing osteoblast precursors are brought as perivascular cells, following further differentiation into osteoblasts and ultimately to bone matrix-embedded osteocytes (Maes et al., 2010). Intramembranous ossification, on the other hand, is responsible for the development of flat bones, as well as the lateral half of the clavicle, without the necessity of forming a cartilaginous intermediate template. In this process, mesenchymal progenitors directly differentiate into osteogenic cells (Kanczler and Oreffo, 2008).

### Physiological fracture healing cascade

The process by which bones heal following fracture differs from natural bone formation; however, following fracture fixation, ossification may occur via endochondral and/or intramembranous pathways depending on the vascular supply and stability of the fracture fixation (Dimitriou et al., 2005). The initial stages of bone repair after fracture include the formation of a hematoma secondary to vascular damage to the injury site. Simultaneously, injury to soft tissues is coupled with recruitment of neutrophils to phagocytize microorganisms and tissue debris within the site of injury. This process is initiated via release of multiple cytokines which causes vasodilation and hyperemia at the site of injury and continues for several days during the inflammatory phase until fibrous tissue, and later bone and cartilage formation is initiated during the repair phase (Claes et al., 2012). Following the influx of neutrophils, macrophages entering the injury site continue phagocytizing debris and initiate the repair cascade by promoting an angiogenic response (Wu et al., 2013). Days following the initial injury, granulation tissue begins to replace the hematoma and necrotic tissue is removed via osteoclasts. Revascularization of the injured site not only re-establishes normoxic conditions and help removing debris, but is also necessary for the recruitment of mesenchymal osteochondroprogenitor cells (Marsell and Einhorn, 2011). After 2–3 weeks post-injury, hematoma is replaced with extracellular matrix formed by fibroblasts and chondroblasts derived from the recruited progenitor cells, developing the soft callus that is characterized by decreased motion of soft tissues, pain, and swelling. During this process, intramembranous bone growth begins as progenitor cells are stimulated to directly form osteoblasts at the periosteum and endosteum, ultimately fueling the woven bone formed peripherally. The soft callus is slowly converted into hard callus over the course of 3–4 months as endochondral ossification is initiated within the fracture gap to allow for woven bone formation. This process begins peripherally and progresses centrally as woven bone fills the fracture gap and unites the two ends of the fracture. Following completion of the hard callus, the woven bone is then remodeled into lamellar bone over the course of months to years, which allows for restoration of the canal and its bony properties.

As mentioned before fracture healing is a complex biological process involving a number of phases namely the acute inflammatory response, the recruitment of Mesenchymal Stem cells (MSCs), the generation of cartilaginous/periosteal bony callus, revascularization, mineralization, and resorption of cartilaginous callus, and finally bone remodeling. Within this cascade the homing of resident or systemically mobilized MSCs to the fracture site is of outmost importance and mainly mediated by signaling cascades such as stromal cell-derived factor (SDF)1/CXC chemokine receptor (CXCR) 4 signaling axis (Shirley et al., 2005; Kitaori et al., 2009). Moreover, during bone resorption active TGF-β1 release from bone matrices induces the recruitment of MSCs to the fracture site through SMAD signaling pathway (Tang et al., 2009) whereas MSCs stimulate neo-vessel formation enhancing further the fracture healing cascade (Todeschi et al., 2015). In a delicate review, Herrmann et al. present strategies to enhance MSC homing to the fracture site including local delivery of homing factors, delivery of genes and genetic manipulation of transplanted cells (Herrmann et al., 2015). Overall, MSCs are coupling bone resorption and bone formation processes and their homing augments fracture healing by regulating bone remodeling and neo-angiogenesis phases.

## Bone tissue engineering

**Figure 1:**
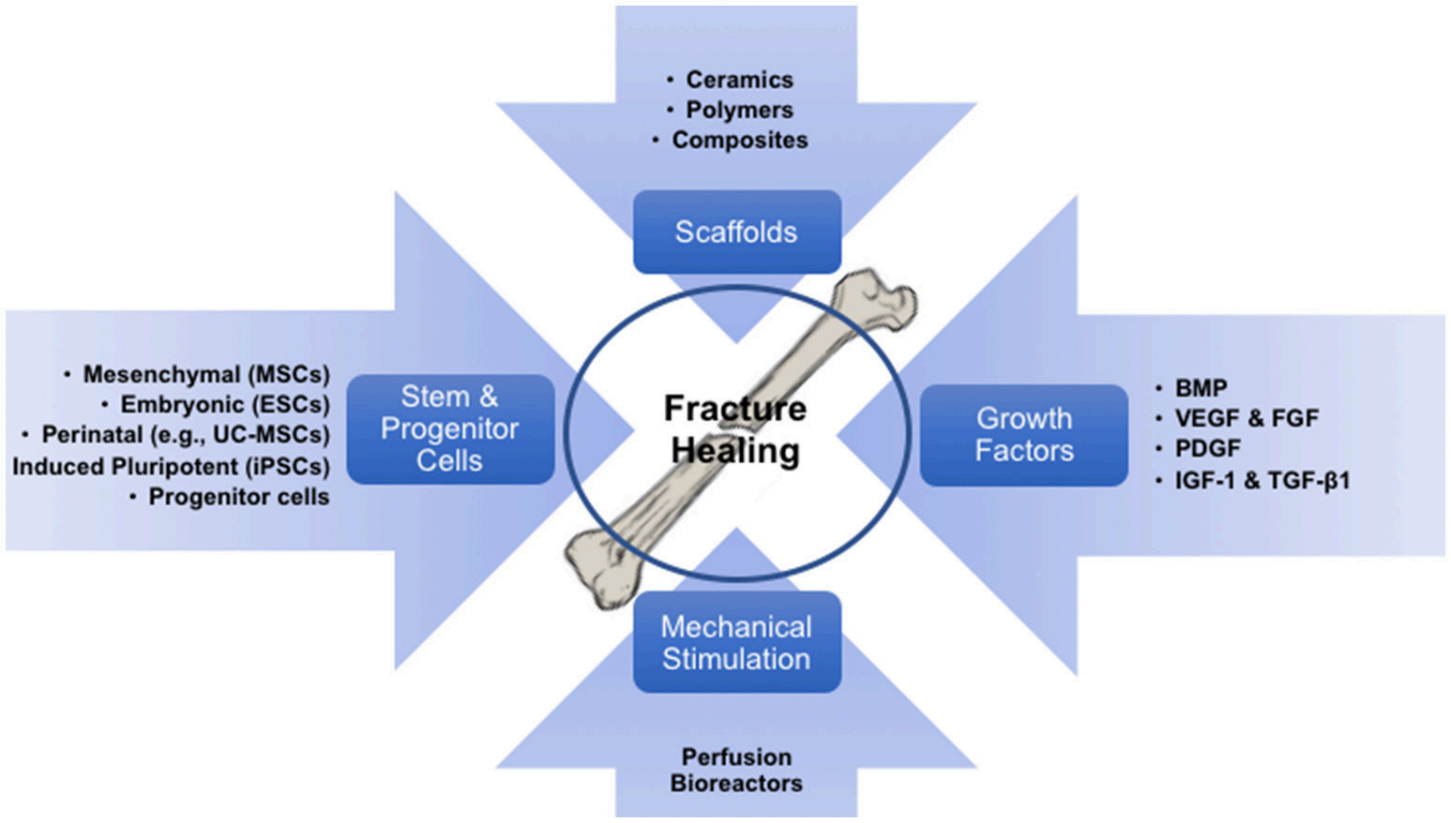
Diagram illustrating the processes which fuels bone tissue engineering, involving its components (cells, biomaterials/scaffolds and growth factors), and the required exposure to mechanical environments to pre-conditioning the engineered implants.

The concept of BTE involves the integration of various concerting components: stem cells held together by a tri-dimensional biomaterial framework which provides the shape and initial mechanical strength, and molecular signals that induce differentiation of progenitor cells into the osteoblastic phenotype. The resulting construct can then be mechanically pre-conditioned *in vitro* to acclimate the growing structure to *in vivo* conditions, thus improving the functional coupling to the host bone (Petite et al., 2000). Here, we review the four fundamental components that take part in BTE, specifically: stem cells, biomaterials, growth factors/morphogens, and mechanical stimulation (Figure 1).

### Stem cells

Tissue-specific cells (e.g., osteoblasts) can be used as the cellular component of engineered bone implants. However, technical difficulties associated with their harvesting, expansion into meaningful numbers and phenotypic maintenance undermine the benefits of using primary cells. Consequently, various types of stem cells have been largely proposed as a viable and easy source of osteoblast progenitors during the creation of engineered bone implants.

#### Mesenchymal stem cells

Mesenchymal stem cells (MSCs) are multipotent adult stem cells that exhibit great differentiation potential into many different types of tissue lineages, including bone (osteoblasts), cartilage (chondrocytes), muscle (myocytes), and fat (adipocytes). Adult MSCs act as an inducible reserve force for tissue regeneration after injury (Caplan and Correa, 2011a,b), and therefore have been studied extensively for their therapeutic potential in fracture healing and bone regeneration. MSCs can be isolated from many different tissues including bone marrow, skeletal muscle, synovial membrane, and adipose tissue. There has consequently been substantial research regarding the osteogenic potential of MSCs obtained from different tissue sites.

Bone marrow-derived stem cells (BMSCs) are currently the most commonly utilized and researched source of adult mesenchymal stem cells due to their relatively easy harvesting, high proliferative capacity, and established regenerative potential (Baksh et al., 2007). Various animal models of clinically significant bone defects have shown that a cell-based therapy with allogenic BMSCs grafts is effective in regenerating bone, providing evidence for a viable alternative to autologous bone transplants (Jones et al., 2016). Studies have found BMSCs to be more efficient at differentiating into osteoblasts compared to adipose-derived MSCs (ADSCs) (Han et al., 2014). Cultured-expanded BMSCs have also been used in large cohort clinical trials showing no complications in long-term follow-up. In early clinical trials, autologous cultured BMSCs were seeded on ceramic biomaterials to treat large bone segmental defects. Local implantation at the defect site of 2.0 × 10^7^ MSCs per ml resulted in complete fusion at 5–7 months post-surgery. Most importantly, 6–7 years follow-up showed that good integration was maintained with no further fractures (Marcacci et al., 2007). In a large clinical trial consisting of 64 patients, various long bone fractures have been treated by local injection of 3.0 × 10^7^ osteogenically differentiated autologous BMSCs per ml mixed with fibrin. Two months follow-up, osteoblast injection showed no complications and significant fracture healing acceleration (Kim et al., 2009). Interestingly, Zhao et al. showed that early stage osteonecrosis of femoral head can be treated by local injection of 2.0 × 10^6^ autologous BMSCs (Zhao et al., 2012). No complications were observed whereas 5 years follow-up only 2 of 53 BMSC-treated femoral heads progressed and underwent vascularized bone grafting. Upper limb non-unions have been also treated in 8 patients using 0.25–1.0 × 10^6^ osteogenically differentiated autologous BMSCs per ml in fibrin clot constructs. Up to 6 years follow-up no complications were observed whereas all patients recovered limb function (Giannotti et al., 2013). Overall, the current body of literature provides support for the viability and utility of BMSCs in the clinical setting of bone defects. However, limitations regarding BMSCs cell yields during harvest, especially in older patients (Mareschi et al., 2006), the requirement of expansion when used alone (not as part of BMAC), the proven reduced regenerative ability with extended expansions (Both et al., 2011) and an increased patient morbidity and risk related to the increased number of surgical procedures all necessitate the need for further research into alternative MSCs harvest sites.

Minimal manipulation of BM is an alternative way to treat various bone defects by using non-cultured, heterogeneous, point-of-care BM aspirate concentrate (BMAC) or “viable allografts.” Bone marrow concentrates are originating from density gradient centrifugation and their MSC content is usually evaluated based on *in vitro* CFU-F capacity (Woodell-May et al., 2015). In contrast, viable allografts are extracted from cadaveric cancellous bone after the selective removal of the immune cell component from the graft, while preserving the MSC fraction (Baboolal et al., 2014). On this basis, Hernigou et al. have used BMAC to treat various orthopedic complications including avascular necrosis, fracture nonunion and rotator cuff repair (reviewed in Jones et al., 2016). Interestingly, they looked at diabetic patients with ankle nonunion, comparing autologous BMSC-containing BMAC with standard bone iliac crest autograft. They found that 82.1% of BMAC-treated patients showed nonunion healing with minimal complications, while only 62.3% in autograft-treated patients showed nonunion healing with major complications observed (Hernigou et al., 2015).

Another readily available source of MSCs under investigation is adipose tissue as it can be easily isolated from plastic surgery or biopsies. The stromal vascular fraction (SVF) derived from adipose tissue is composed of heterogenous cell populations including adipose-derived stem cells (ADSCs), vascular endothelial cells, vascular smooth muscle cells, preadipocytes, and haematopoietic origin cells (Zuk et al., 2001, 2002; Daher S. R. et al., 2009). Although direct grafting of ADSCs has not demonstrated much success in healing critical sized bone defects (Peterson et al., 2005), there is increasing interest in applying osteoinductive factors to ADSCs in the hopes of enhancing osteogenesis. A study by Di Bella et al. demonstrated bone regeneration in rabbit critical-sized skull defects treated with autologous, osteogenically-induced ADSCs grafted onto fibronectin-coated polylactic acid biomaterials (Di Bella et al., 2008). Another study demonstrated repair of a cranial bone defect in canine models using osteogenically-induced ADSCs grafted onto a coral biomaterial (Cui et al., 2007). A recent paper by Fan et al. showed enhancing osteogenic differentiation of ADSCs through treatment with phenamil, a positive regulator of BMP signaling, along with suppression of the BMP antagonist noggin through gene manipulation (Fan et al., 2016). Interestingly, two clinical studies combining ADSCs with specialized biomaterials (Mesimäki et al., 2009) or autologous bone grafting (Lendeckel et al., 2004) indicated bone reconstruction *in vivo*. In a case study, Mesimaki et al. performed maxillary reconstruction using a microvascular flap construct generated by combining autologous ADSCs, β-tricalcium phosphate and bone morphogenetic protein-2. At 36 months follow-up the implants were osteo-integrated without any adverse effects and the defect was fully reconstructed (Mesimäki et al., 2009). In another case study, Lendeckel et al. treated severe calvarial defects using autologous ADSCs in fibrin glue combined with autologous cancellous bone graft. At 3 months follow-up, new bone formation and near complete calvarial continuity were observed (Lendeckel et al., 2004). Collectively, these studies demonstrate novel methods of enhancing bone formation using ADSCs, providing promising evidence for the potential therapeutic role ADSCs could play in BTE. However, further studies are required to assess and verify the safe outcome of the clinical procedures using *in vitro* cultured ADSCs.

Several other tissues contain MSCs, including synovial membrane and skeletal muscle, all of them sharing a perivascular phenotype as pericytes (Caplan, 2008; Crisan et al., 2008). Synovial membrane-derived MSCs (SMSCs) are multipotential stromal cells that provide a good therapeutic alternative to tissue engineering protocols for focal cartilage injuries (Ogata et al., 2015; Kubosch et al., 2018). However, previous studies showed that MSCs derived from normal or OA synovium have the potential to differentiate toward osteogenesis *in vitro* (De Bari et al., 2001; Sakaguchi et al., 2005). Interestingly in a recent study, Hatakeyama et al. showed that SMSCs derived from knee joints have superior osteogenic and adipogenic capacity than SMSCs derived from hip joints (Hatakeyama et al., 2017). However, another study showed that SMSCs have inferior osteogenic capacity from periosteum-derived MSCs (De Bari et al., 2008). Therefore, further preclinical studies have to be performed to evaluate SMSCs' applicability in bone regeneration. On the other hand, current literature suggests that skeletal muscle-derived MSCs (SMDCs) are able to differentiate into several different types of mesenchymal lineages and show potential for *in vivo* bone regeneration when combined with osteoinductive biomaterials (Owston et al., 2016). Many previous studies have successfully demonstrated SMDCs' osteogenic potential *in vitro* (Jackson et al., 2011; Gao et al., 2013, 2014; Downey et al., 2015). However, Sakaguchi et al. indicated lower osteogenic potency for SMDCs compared to matched BM, synovium and periosteum MSCs (Sakaguchi et al., 2005). Similar results were obtained from Jackson et al. showing lower amounts and significantly lower ALP upregulation in SMDCs compared to BMSCs (Jackson et al., 2011). Recently, Miao et al. were able to induce a 24-fold increase in alkaline phosphatase activity (an early marker for early osteogenesis) in skeletal muscle discs cultured in osteogenic medium, with demonstrable calcified mineral deposits appearing on the superficial layers of the muscle discs after 8 weeks of osteoinduction. This study demonstrated the potential role of skeletal muscle in expedited BTE and provides support for further investigation of this tissue site for MSCs extraction (Miao et al., 2017).

One tissue site just recently being explored for musculoskeletal bone regeneration is dental pulp-derived stem cells (DPSCs). DPSCs were first isolated and characterized by Gronthos et al. within the dental pulp core and found to have similarities to BMSCs (Gronthos et al., 2000). DPSCs have the advantage of being relatively easy to harvest with very low rates of morbidity compared to BMSCs (Graziano et al., 2008). In addition, they can be safely cryopreserved and possess extensive differentiation ability into adipogenic, chondrogenic, and osteogenic lines (Graziano et al., 2008). Specifically, DPSCs have been successfully differentiated toward osteogenesis in both *in vitro* and *in vivo* settings indicating increased expression of bone-related markers and newly bone formation (Lindroos et al., 2008). In a pioneering study, d'Aquino et al. showed that DPSCs synergistically differentiate into osteoblasts and endotheliocytes *in vitro* whereas their transplantation in immunocompromised rat model result in the formation of bone tissue structure with an integral blood supply similar to adult human bone structure (d'Aquino et al., 2007). Kanafi et al. indicated the increased DPSC osteogenic capacity in alginate microsphere platform by observing enhanced mineralization and upregulated levels of osteo-related genes. Interestingly, the same group showed that DSPCs immobilization in alginate microspheres initiate their osteogenic differentiation without any medium induction (Kanafi et al., 2013). Recently, Akkouch et al. demonstrated successful extracellular mineralization of osteoblast-like cells derived from DPSCs seeded onto collagen-hydroxyapatite-poly(l-lactide-co-ϵ-caprolactone) matrix (Akkouch et al., 2014). In a rat calvarial critical-sized defect model, Maraldi et al. showed that DPSCs embedded in collagen sponges result in newly bone formation at 4 week and in almost complete bringing of the defect at 8 week of cranial implantation (Maraldi et al., 2013). Despite the potential of DPSCs in BTE, very few clinical trials have been reported so far and further research is needed to assess their applicability for clinical application. According to the previous studies, DPSCs' combination with specialized biomaterials can harness their increased osteogenic capacity for effective bone reconstruction clinical applications.

Finally, perinatal tissues such as umbilical cord blood (UCB), umbilical cord tissue (UC) and placenta have been considered as alternative sources of MSCs. Moreover, UC and UCB are readily available sources of allogeneic MSCs for therapeutic applications due to the worldwide existence of both public and private cord blood (CB) stem cell banks. In various preclinical models, CB-derived MSCs have successfully enhanced bone regeneration in conditions of non-systemic and systemic bone loss (Guillot et al., 2008; Jäger et al., 2009; Liu et al., 2009). Although, CB-derived MSCs are considered to circulate in preterm fetus blood, their isolation is not consistent (Secco et al., 2009). In contrast, MSCs can be easily isolated from various compartments of UC tissue including Wharton's Jelly, umbilical vein and umbilical artery areas (Kouroupis et al., 2013). Unlike ESCs, there are no reports of UC MSC teratoma formation capacity whereas studies showed that compared with BM MSCs and ESCs, UC MSCs show a gene expression profile similar to that of ESCs and faster self-renewal capacity from BM MSCs (Hsieh et al., 2010; Fong et al., 2011). In a previous study, Mennan et al. compared MSCs from all different UC tissue regions and indicated that Wharton's Jelly MSCs show the best osteogenic differentiation capacity (Mennan et al., 2013). *In vivo*, UC MSCs have been successfully used to treat critical-sized craniofacial defects in rat model (Chen et al., 2013). Chen et al. compared the regenerative capacity of UC MSCs and BM MSCs combined with RGD-modified macroporous calcium phosphate cements (CPC) in a critical-sized athymic rat pariental bone defect model. Moreover, 24 weeks post-implantation UC MSC-CPC and BM MSC-CPC groups showed similar high bone mineral density, new bone amount and vessel density (Chen et al., 2013). Importantly, in a clinical study allogeneic UC MSCs have been successfully used to treat infected nonunion of large bone segmental defect (Dilogo et al., 2017).

Overall, compared to ESCs, perinatal stem cells show crucial benefits for effective therapeutic applications. Perinatal cells are isolated from a formerly discarded tissue that has unlimited availability whereas their collection, preparation and application is not raising any ethical issues (Watson et al., 2015). Additionally, perinatal cells share stemness markers with both ESCs and MSCs, they are not tumorigenic and they are hypoimmunogenic (Kim et al., 2013).

#### Embryonic stem cells

First derived in 1998 from human blastocysts, pluripotent human embryonic stem cells (hESCs) maintain the developmental potential for all three embryonic germ layers even after months of *in vitro* proliferation (Thomson et al., 1998), thus demonstrating a potential source for tissue engineering-based therapies. Successful differentiation of hESCs into the osteogenic lineage has been demonstrated in numerous studies both *in vitro* and *in vivo* (Tang et al., 2012; Taiani et al., 2014). A number of researchers have developed ways to derive hMSCs from hESCs that are morphologically and phenotypically similar to BMSCs without the use of a feeder layer (Olivier et al., 2006), providing an alternative and plentiful source of reproducible and more embryonic-like hMSCs. There have also been multiple studies demonstrating that hESCs treated with osteogenic factors will undergo differentiation toward an osteogenic lineage. In fact, after osteogenic induction, hESCs have been shown to possess molecular and structural features resembling bone tissue by the formation of mineralized bone nodules *in vitro* (Woll et al., 2006; Arpornmaeklong et al., 2010). Although several advantages have been discovered concerning the use of hESCs, potential limitations exist regarding the use of pluripotent cells secondary to their unexpected differentiation, especially their link to teratoma formation (Cunningham et al., 2012).

Related to tissue engineering, one *in vitro* study was able to induce attachment, proliferation, and bone mineral synthesis of hESC-derived MSCs on a novel calcium phosphate cement-chitosan-RGD biomaterial (Chen et al., 2013). Kim et al. demonstrated significant *in vivo* bone formation in immunodeficient mice by subcutaneously seeding osteogenic cells derived from ESCs onto a three-dimensional porous poly (d,l-lactic-co-glycolic acid)/hydroxyapatite composite biomaterial (Kim et al., 2008). Both of these studies provide evidence in favor for the proliferative and osteogenic compatibility of ESCs with various engineered biomaterials and a secondary functional secretion of bone matrix. Interestingly, though osteogenic cultures of ESCs are generally derived from embryoid bodies, one study found that culturing hESCs without going through that stage led to a seven-fold increase in the number of osteogenic cells produced, as well as spontaneous bone nodule formation much sooner than cells differentiated from embryoid bodies directly (Karp et al., 2006).

Despite the excitement generated by their enormous potential for proliferation and differentiation, hESCs have several limitations that must be further investigated. Challenges concerning the complicated conditions required to culture hESCs, including the feasibility and viability of using feeder layers, the danger of teratoma formation and immune reactions, as well as the surrounding ethical, religious and moral debate, all pose challenges to the role of hESCs as active participants of regenerative medicine-based clinical protocols (Richards et al., 2003; de Miguel-Beriain, 2015).

#### Induced pluripotent stem cells

Induced pluripotent stem cells (iPSCs), which are derived and reprogrammed directly from adult somatic cells (e.g., skin fibroblast), have the ability to give rise to every type of cell in the body and to propagate indefinitely. They were first developed by Takahashi and Yamanaka in 2006, through the lentiviral-based introduction of a defined set of reprogramming transcription factors (c-Myc, Oct3/4, Sox2, and Klf4), which induced a pluripotent state comparable to ESC. Consequently, iPSCs hold enormous potential for the entire field of regenerative medicine, as they possess a comparable pluripotency and differentiation potential as hESCs (Takahashi and Yamanaka, 2006), yet avoid immune rejection since they are derived from the patient's own cells (Im, 2015). In addition, given that the generation of iPSCs bypasses the use of human embryos, that provides a potential answer to the ethical dilemma surrounding ESCs.

iPSCs generated through embryoid bodies have been shown to generate MSC-like cells *in vitro* that have the potential of further differentiating into osteoblasts (Li et al., 2010), while also demonstrating osteogenic potential comparable to that of BMSCs *in vivo* (Ko et al., 2014). Additionally, animal studies have demonstrated that MSC-like cells cultured from iPSCs have the capacity to form mature mineralized material that is histologically similar to bone (Hynes et al., 2013). In addition to a low efficiency during the generation of iPSCs, the potential formation of teratomas is of clinical concern. Levi et al. demonstrated the *in vivo* differentiation and *de novo* bone formation of iPSCs without the formation of a teratoma when grafted onto a hydroxyapatite-coated, BMP-2-releasing poly-L-lactic acid biomaterial (Levi et al., 2012). Similarly, Kang et al. demonstrated the first direct differentiation of human iPSCs (hiPSCs) into functional osteoblasts that subsequently went on to deposit calcified bone matrix. By using adenosine to induce osteogenesis of hiPSCs, they were able to demonstrate that the hiPSC-derived osteoblasts participated in the healing of critical sized bone defects without the formation of a teratoma (Kang et al., 2016). In a recent pioneering study, Kouroupis et al. generated a bioartificial ACL graft *ex vivo* by simultaneously differentiating MSC-like cells cultured from iPSCs toward bone and ligament at the ends and central part of a biomaterial using either BMP-2/FGF-2 or TGF-β/FGF-2 growth factor combinations, respectively. ACL graft *in vivo* implantation in a swine ACL injury model resulted in superior morphological and biochemical ACL tissue formation for both the bony and ligamentous parts of the reconstructed tissue (Kouroupis et al., 2016). Though the area of iPSC research is still new, taken together, these findings indicate the exciting promise iPSCs hold for the future of osteogenic tissue engineering. Nevertheless, further clinical investigation focusing not only on efficacy (e.g., osteogenic potential) but also safety (e.g., teratoma formation) becomes paramount before accepting iPSCs as a viable therapeutic option.

Finally, iPSC-based therapy brings additional challenges such as the technical and logistical issues related with their generation. Tissue sourcing, manufacturing protocols, required expansion, systematic testing and quality control, validation, and storage constitute technical aspects that impact the costs associated the generation of these “off the shelf” products, delaying their translation into potential clinical therapies. In order to advance the field toward standardization, as product reproducibility need to meet specific standards, a number of consortiums and forums (e.g., CCRM, CIRM, HiPSCi, STemBANCC, ISCF, and ISCBI) have been established to support with best practices to generate and supply hiPSCs lines for basic and potential clinical research. Consequently, manufacturers have initiated projects aiming at generating iPSC products based on strict cGMP-compliant conditions to overcome some of these challenges, involving best practices for cell culture, documentation and quality control (Stacey et al., 2013).

#### Progenitor cells

Endochondral BTE using chondrocytes and chondroprogenitors has been recently reported by various groups. Two independent studies showed that both human (Narcisi et al., 2011) and porcine (Jeong Claire et al., 2012) articular chondrocytes can be induced toward endochondral ossification and produce a hypertrophic phenotype upon induction with TGFβ-1 and BMP-2, respectively. Although, *in vitro* TGFβ-1-expanded cells showed strong mineralisation and impaired p38 kinase activity which is related to chondrocyte differentiation, their *in vivo* ectopic implantation with ceramic biomaterial failed to reach overt ossification (Narcisi et al., 2011). Also, Jeong et al. showed that seeded chondrocytes on BMP-2-loaded polycaprolactone biomaterials when subcutaneously implanted *in vivo* can result in bone formation but only at the periphery of the biomaterial (Jeong Claire et al., 2012). Therefore, the use of mature chondrocytes for BTE may be limited due to their mature phenotype, reduced *in vitro* expansion and the risk of arthritic development. In contrast, others showed that chondrocyte-like progenitors possessing a transient phenotype *in vitro*, can be effectively induced toward endochondral bone formation in *in vivo* settings (Oliveira et al., 2008; Weiss et al., 2012). On this basis, studies showed that MSCs induced for 21 days toward chondrogenesis *in vitro*, with the common TGF-β based chondrogenic cocktail, are gradually obtaining a transient hypertrophic phenotype that can result in subsequent bone formation upon implantation *in vivo* (Vinardell et al., 2012; Giavaresi et al., 2013). Interestingly, extended induction for 7 additional days with β-glycerol-phosphate (Wang et al., 2011) or for 14 additional days with β-glycerol-phosphate and thyroxine (Scotti et al., 2013) result in increased bone formation *in vivo*. Further, examples of tissue-engineered cartilage constructs that can result through endochondral progression to bone formation *in vivo* are reviewed in Thompson Emmet et al. (2014). However, using mature metabolically active hypertrophic cells may not be advantageous as they show limited bioactive lifespan compare to the high molecular plasticity of MSCs in activating pathways related to endochondral bone formation process.

### Biomaterials

It is now nearly 50 years since Professor Hench in 1969 introduced the term “bioactivity” in biomaterials field, which is the characteristic chemical bonding between biomaterials and cells (Hench, 2006). Specifically, the function of the biomaterial in BTE is to serve as a tri-dimensional framework for the stem cells to attach, grow and differentiate. There are several components of the biomaterial required for successful incorporation and functionality, including: (1) biocompatibility: incorporation into host tissues without eliciting an immune response; (2) biodegradability: as bone replaces the biomaterial, it provides supportive mechanical properties to withstand loading forces and uniformly distribute stresses; (3) proper surface properties and porosity: to influence cellular proliferation and differentiation; and (4) osteoinductive and osteoconductive properties: to recruit osteoprogenitors to the defect region and provide a controlled release of differentiation cues (Liu et al., 2013). Here we focus on three categories of biomaterials commonly used: ceramics, biodegradable polymers, and composite biomaterials. The applicability of various biomaterials combined with MSCs for bone segmental defects treatment in preclinical settings is presented in Table 1.

**Table 1:** Preclinical studies using MSCs and biomaterials for the treatment of bone segmental defects.

**References**	**Cells**	**Biomaterials**	**Animal Model**	**Outcome**
Bruder et al. (1998)	Canine BMSCs (7.5 × 10^6^/ml)	Three groups used: 1) HA-TCP-BMSCs, 2) HA-TCP, 3) Untreated	Segmental femoral bone defect (2.1 cm) in canine model	- At 16 weeks, radiographic union was established rapidly at the interface between the host bone and the HA-TCP-BMSCs implants only - Both woven and lamellar bone had filled the pores of the HA-TCP-BMSCs implants
Kon et al. (2000)	Ovine BMSCs (2.5 × 10^5^/ml)	Two groups used: 1)HA-BMSCs 2) HA	Segmental tibial bone defect (3.5 cm) in ovine model	-At 2 months, extensive bone formation in HA-BMSCs implants within the macropore space and around the implant - Stiffness higher in HA-BMSCs implant/bone complex compared to HA control group
Arinzeh et al. (2003)	Canine BMSCs (7.5 × 10^6^/ml)	Three groups used: 1) HA-TCP-allogeneic BMSCs 2) HA-TCP 3) Untreated	Segmental femoral bone defect (2.1 cm) in canine model	- No lymphocytic infiltration occurred and no antibodies against allogeneic cells were detected - At 16 weeks, new bone had formed throughout the HA-TCP-allogeneic BMSCs implant
Bensaïd et al. (2005)	Ovine BMSCs (1 × 10^7^/ml)	Four groups used: 1)Coral HA-BMSCs 2) Coral HA 3) Autologous bone graft 4) Untreated	Segmental metatarsus bone defect (2.5 cm) in ovine model	- At 4 months, coral HA-BMSCs implants show the same amount of newly formed bone - with autologous bone and at 14 months are completely replaced by newly formed, structurally competent bone
Viateau et al. (2007)	Ovine BMSCs (8.28 ± 1.32 × 10^6^/implant)	Three groups used: 1) Coral-BMSCs 2) Coral 3) Untreated	Segmental metatarsus bone defect (2.5 cm) in ovine model	-At 6 months, radiographic, histological, and computed tomographic tests performed showed that the osteogenic abilities of the coral-BMSCs implants were significantly greater than those of coral scaffold alone
Zhu et al. (2006)	Caprine BMSCs (20 × 10^6^/ml)	Two groups used: 1) Coral-BMSCs 2) Coral	Segmental femoral bone defect (2.5 cm) in caprine model	-At 4 months bony union was observed in coral-BMSCs implant and engineered bone was further remodeled into newly formed cortexed bone at 8 months
Mastrogiacomo et al. (2007)	Ovine BMSCs (0.5–1.0 × 10^8^/ml)	Two groups used: 1) Si-TCP-BMSCs 2) Si-TCP	Segmental tibial bone defect (4 cm) in ovine model	- At 4 months, 4 out of 5 animals implanted with Si-TCP-BMSCs implants, a progressive new bone formation, from the osteotomy defect edge toward the implant mid zone, was observed - Neither bone formation nor scaffold resorption was observed in Si-TCP group
Liu et al. (2008)	Caprine BMSCs (2 × 10^7^/ml)	Three groups used: 1) β-TCP-BMSCs 2) β-TCP 3) Untreated	Segmental tibial bone defect (2.6 cm) in caprine model	-At 32 weeks, bony union can be observed at β-TCP-BMSCs group by gross view, X-ray and micro-computed tomography detection, and histological observation - In β-TCP-BMSCs group the implants are almost completely replaced by tissue-engineered bone whereas bone mineral density is significantly higher than in β-TCP group
Giannoni et al. (2008)	Ovine BMSCs (70–100 × 10^6^)	Three groups used: 1) HA-Si-TCP-BMSCs 2) HA-Si-TCP 3) Autologous bone graft	Segmental tibial bone defect (4.5 cm) in ovine model	-At 20–24 weeks, autologous bone graft group performed best -as assessed radiologically - In other groups very limited healing was detected whereas a partial bone deposition occurred at the periphery of the bony stumps only in HA-Si-TCP-BMSCs group
Nair et al. (2008)	Caprine BMSCs (1 × 10^5^/cm2)	Two groups used: 1)HASi + BMSCs 2) HASi	Segmental femoral bone defect (2 cm) in caprine model	-At 4 months, both HASi + BMSCs and HASi implants showed good osteointegration and osteoconduction - The superior performance of HASi + BMSCs implant was evident by the lamellar bone organization of newly formed bone throughout the defect together with the degradation of the material
Niemeyer et al. (2010)	Human and Ovine BMSCs (2 × 10^7^/ml)	Three groups used: 1)HA-COL-human BMSCs 2) HA-COL-ovine BMSCs (allogeneic) 3) Untreated	Segmental tibial bone defect (3 cm) in ovine model	- At 26 weeks, radiology and histology demonstrated significantly better bone formation in HA-COL-ovine BMSCs group compared to HA-COL-human BMSCs and untreated groups
Nair et al. (2009)	Caprine BMSCs (1 × 10^5^ cm2)	Three groups used: 1)HASi + BMSCs 2) HASi + BMSCs + PRP 3) HASi	Segmental femoral bone defect (2 cm) in caprine model	-At 2 months, in HASi + BMSCs and HASi + BMSCs + PRP groups 60–70% of the mid region of the defect was occupied by woven bone, in line with material degradation
Zhu et al. (2010)	Caprine BMSCs (5 × 10^7^/ml)	Two groups used: 1)Coral-BMSCs 2) Coral-AdBMP-7- BMSCs	Segmental femoral bone defect (2.5 cm) in caprine model	-Much callus was found in the coral-AdBMP-7- BMSCs group, and nails were taken off after 3 months of implantation, indicating that regenerated bone in the defect can be remodeled by load-bearing, whereas this happened after 6 months in the coral-BMSCs group
Cai et al. (2011)	Canine BMSCs (20 × 10^6^/ml)	Four groups used: 1) Coral HA-BMSCs 2) Coral HA-BMSCs (vascularized) 3) Coral HA (vascularized) 4) Coral HA	Segmental fibula bone defect (1 cm) in canine model	- At 3 months, vascularization improved 2-fold bone formation compared to non-vascular group
Reichert et al. (2012)	Ovine BMSCs (35 × 10^6^ cells/250 μl) BMP-7 (3.5 mg/implant)	Five groups used: 1)mPCL-TCP-BMSCs + PRP 2) mPCL-TCP-BMP-7 3) mPCL-TCP 4) Autologous bone graft 5) Untreated	Segmental tibial bone defect (3 cm) in ovine model	- At 12 months, biomechanical analysis and microcomputed tomography imaging showed significantly greater bone formation and superior strength for the biomaterial loaded with rhBMP-7 compared to the autograft
Manassero et al. (2013)	Ovine BMSCs (7.5 ± 1.2 × 10^6^/implant)	Two groups used: 1) Coral-BMSCs 2) Coral	Segmental metatarsus bone defect (2.5 cm) in ovine model	-At 6 months, coral-BMSCs implants showed 2-fold increase in bone formation compared to coral alone
Berner et al. (2013)	Ovine BMSCs (35 × 10^6^/500 μl)	Four groups used: 1)mPCL-TCP-BMSCs (autologous) 2) mPCL-TCP-BMSCs (allogeneic) 3) mPCL-TCP 4) Autologous bone graft	Segmental tibial bone defect (3 cm) in ovine model	-At 12 weeks radiology, biomechanical testing and histology revealed no significant differences in bone formation between the autologous and allogenic mPCL-TCP-BMSCs groups - Both cell groups showed more bone formation than the biomaterial alone
Fan et al. (2014)	Non-human primate BMSCs (5 × 10^6^/implant)	Five groups used: 1) TCP-β-BMSCs 2) TCP-β-BMSCs-fascia flap 3) TCP-β-BMSCs-saphenous vascular bundle 4) TCP-β 5) Untreated	Segmental tibial bone defect (2 cm) in non-human primate model	-At 4, 8, and 12 weeks, the TCP-β-BMSCs-saphenous vascular bundle group could augment new bone formation and capillary vessel in-growth. It had significantly higher values of vascularization and radiographic grading score compared with other groups.
Yoon et al. (2015)	Canine ADMSCs (1 × 10^6^/50 μl)	Five groups used: 1) ASA-ADMSCs 2) ASA-β-TCP-ADMSCs 3) ASA-β-TCP 4) ASA 5) Untreated	Segmental ulna bone defect (1.5 cm) in canine model	- At 16 weeks, histomorphometric analysis showed that ASA biomaterials with ADMSCs had significantly greater new bone formation than other groups
Berner et al. (2015)	Ovine BMSCs (100 × 10^6^)	Three groups used: 1) PCL-HA-allogeneic BMSCs 2) PCL-HA 3) Autologous bone graft	Segmental tibial bone defect (3 cm) in ovine model	- Minimally invasive percutaneous injection of allogeneic BMSCs into biodegradable composite biomaterials 4 weeks after the defect surgery led to significantly improved bone regeneration compared with preseeded biomaterial/cell and biomaterial-only groups
Masaoka et al. (2016)	Non-human primate BMSCs (1.3–4.1 × 10^6^/ml)	Two groups used: 1) β-TCP-BMSCs 2) β-TCP	Segmental femoral bone defect (5 cm) in non-human primate model	-At 8–15 months, five of the seven animals treated with β-TCP-BMSCs implant showed successful bone regeneration
Smith et al. (2017)	Ovine BMSCs (1 × 10^7^/implant)	Three groups used: 1) PLLA-PCL-BMSCs 2) PLLA-PCL 3) Untreated	Segmental tibial bone defect (3.5 cm) in ovine model	-At 12 weeks, both PLLA-PCL-BMSCs and PLLA-PCL groups showed enhanced quantitative bone regeneration - Significant bone regeneration was evident only in the PLLA-PCL-BMSCs group whereas complete defect bridging was not achieved in any group
Berner et al. (2017)	Ovine MPCs, mOB, tOB (35 × 10^6^ cells)	Four groups used: 1)mPCL-TCP-PRP 2) mPCL-TCP-allogenic-MPC 3) mPCL-TCP-allogenic-mOB 4) mPCL-TCP-allogenic-tOB	Segmental tibial bone defect (3 cm) in ovine model	-At 6 months, mPCL-TCP-allogenic-MPC group showed a trend toward a better outcome in biomechanical testing and the mean values of newly formed bone

*BMSCs, bone marrow tissue-derived MSCs; ADMSCs, adipose tissue-derived MSCs; MPCs, mesenchymal progenitor cells; tOBs, axial skeleton osteoblasts; mOBs, orofacial skeleton osteoblasts; PRP, platelet rich plasma; HA, hydroxyapatite; HA-TCP, hydroxyapatite-tricalcium phosphate; HA-COL, hydroxyapatite-collagen; Coral HA, coral hydroxyapatite; HASi, triphasic ceramic-coated hydroxyapatite; Si-TCP, silicon stabilized tricalcium phosphate; mPCL-TCP, medical grade polycaprolactone-tricalcium phosphate; ASA, autologous serum-derived albumin; PCL-HA, polycaprolactone-hydroxyapatite; PLLA-PCL, poly(L-lactic acid)-poly(ε-caprolactone); AdBMP-7, adenovirus mediated bone morphogenetic protein 7*.

#### Ceramics

Known for their effective biocompatibility, ceramic biomaterials are used more commonly in compressive loading conditions as they have very low wear rates due to their high hardness values. However, ceramics are also highly brittle. Because of their properties, ceramic-based biomaterials are commonly used on articulating surfaces, with calcium phosphate (CaP) and tricalcium phosphate being the most common.

In one study, dense calcium sulfate (D-CaS), ultraporous tricalcium phosphate (beta-TCP) and porous silicated calcium phosphate (Si-CaP) biomaterials were used in rabbits. They observed rapid resorption of D-CaS, which left the defect site empty prior to new bone formation. High rates of dissolution have been found to cause biological reactions by inducing inflammation (Lu et al., 2004), as was noted in the D-CaS group. Although both the Si-CaP and beta-TCP biomaterials support early bone apposition, beta-TCP produced an inflammatory response which impaired and reversed bone apposition. Ultimately, Si-CaP biomaterials were found to allow for stable bone apposition with new bone and resorption of the biomaterial with local loads, facilitating production of functional bone at the defect site (Hing et al., 2007). Although beta-TCP impaired bone apposition, other studies have shown it to be an effective biomaterial. Kondo et al. demonstrated purified beta-tricalcium phosphate has the potential for good biocompatibility as bone formation and resorption starts at an early stage after implantation in rat femoral condyles. They noted new bone formation after day 7 and bone marrow within the implanted region by day 28 (Kondo et al., 2005).

Although CaP-based biomaterials have been demonstrated to contain important properties required for BTE such as bioactivity and biocompatibility, their use is limited by their stiffness and low osteoinductivity. Studies have shown this can be resolved with addition of recombinant human bone morphogenetic proteins (rhBMPs) which allow for the potential of bone formation similar to that of autografts, as well as excellent stiffness (Sun and Yang, 2015). CaP-based biomaterials have proven to be an effective ceramic biomaterial –however, porous hydroxyapatite (HA) scaffolds have also shown promising results in improving critical sized long bone defects. In a study by Maracci et al. four patients with large diaphyseal defects were treated with porous HA biomaterials seeded with bone marrow stroma. They reported complete fusion between the implant and host bone between 5 and 7 months post-surgery and good integration of implants after long-term follow-up (Marcacci et al., 2007). As extensively reviewed by Habraken et al. (2016) osteoinduction has been demonstrated in preclinical settings for various CaP phases including HA, TCP, biphasic calcium phosphate (BCP), dicalcium phosphate dehydrate (DCPD), dicalcium phosphate (DCPA), carbonated apatite (CA), and osteocalcium phosphate (OCP), and in various formats including cements, coatings, sintered ceramics, and coral-derived ceramics. Material properties essential for rendering a ceramic osteoinductive are its chemical composition, its macrostructure and its surface micro- and nanostructural properties. It is well accepted that enhanced CaP osteoinduction can be achieved by simultaneously increasing *in vivo* material's degradability while keeping relatively stable material's surface for effective ceramic-induced *de novo* bone formation. An important method to enhance the CaP osteoinductivity is through the incorporation of bioinorganic compounds such as strontium ranelate and fluoride, which are usually found in trace amounts in human body but result in accelerated bone formation and improve bone bonding in orthopedic, craniomaxillofacial, and dental applications (Habibovic and Barralet, 2011).

Overall, ceramic biomaterials show good biocompatibility and bioactivity but they demonstrate low toughness and insufficient strength. This is a major disadvantage and as a result, ceramic biomaterials can be used in non- or low-loading orthopedic applications. Nowadays, biomaterial engineering efforts are focused on improving their properties by the use of nanoscale second phase reinforcing (including nanoparticles, nanotubes, and nanosheets), the formation of surface coatings (such as polymers and glasses) (Hee Ay et al., 2014), and the use of self-toughening methods (via microstructure design) (Li et al., 2012).

#### Polymers

Biodegradable polymer biomaterials, including polylactides (PLLA, PDLA), collagen, polyglycolide (PGA), and poly(ε-caprolactone) (PCL), are best known for their capacity to support tissue growth and remodeling prior to being resorbed. These biomaterials are hydrolytically stable as they contain ester bonds in their backbone, specifically short aliphatic chains, which allows them to be used as degradable biomedical constructs (Valappil et al., 2006). Polymers may be natural or synthetic, with each type providing unique properties regarding their biodegradable rates, consistency, predictability, and their ability to interact with host tissues.

Poly (lactic acid) (PLA) has been proven to be an effective polymer as studies have shown it can induce mature osteogenic phenotypes after 4 weeks of cell seeding. PLA-based biomaterials have shown adhesion and growth of human osteoprogenitors cells ultimately leading to bone healing (Yang et al., 2001). In contrast to PLA, PGA polymers have a longer chain synthesis and are not soluble in most organic solvents—however, they are also vulnerable in load bearing areas as they also contain poor mechanical properties and unfavorable surfaces for cell attachment and proliferation product (Kinoshita et al., 2008), limiting its use as a biomaterial. Further research should be done as some studies have shown the potential to improve stem cell adherence by adding bioactive materials. PLGA is another polymer that has been used to generate biomaterials given the weakening integrity of the polymer as bone healing progresses, which allows a flexible adaptation to bone growth (Habal and Pietrzak, 1999). Its use in BTE has been supported when combined with BMSCs, an osteoinductive medium, and specific bioreactor conditions (Koc et al., 2008).

Given its high permeability and thermal stability secondary to its aliphatic, semi-crystalline properties, PCL is the most heavily researched polymer (Woodruff and Hutmacher, 2010). PCL is a favorable biomaterial for BTE, especially in long-term implantable systems as it is known to maintain its mechanical properties for up to 6 months and then gradually degrade over a 2 year period (Calvert et al., 2000). In addition, PCL is FDA approved, easy to manufacture and manipulate for different anatomical locations, and is highly biocompatible, making it a favorable source for biomaterial design. Overall, biodegradable polymer biomaterials are widely used but they show some limitations in orthopedic applications which include being prone to deformation, weak mechanical properties, and failure to integrate strongly with bone as they exhibit different elastic properties to that of both cancellous and cortical bone (Asti and Gioglio, 2014). Specifically, natural polymers show poor thermal stability, processability and degradation control whereas synthetic polymers show poor cell adhesion as they lack bioactivity (Gautam et al., 2013; Asti and Gioglio, 2014). Most importantly, the degradation products of synthetic polymers are mildly acidic causing local reduction of cell growth and non-specific inflammation (Kim et al., 2007; Song et al., 2011). To address these issues, researchers have focused on improving polymer degradation rates, bioactivity and mechanical properties. Similar to ceramic biomaterials, polymers can be reinforced by nanoscale second phase method (Guo et al., 2009). Studies showed that polymer properties can be improved by the formation of composites with bioactive ceramics and/or other polymers (see Composites section) (Choi et al., 2010; Meseguer Olmo et al., 2012). Therefore, degradation rates can be controlled by adjusting the ratios of biomaterials used to generate composites (Wu et al., 2012) whereas biomaterials' acidic degradation products can be neutralized by adding alkaline materials to polymers (Westhauser et al., 2016).

#### Composites

Composite biomaterials consist of polymers combined with ceramics, merging the benefits of both classes while limiting their short-comings. They possess suitable properties for BTE such as mechanical toughness, improved biocompatibility, decreased creep-induced failure, load-bearing capabilities, host-implant interactions, and bioactivity (Niemeyer et al., 2004). By adding metals to these composites, additional benefits can be seen in bone interactions, strength, and osteogenesis. Resorbable polymer composites have been successfully used in oral and maxillofacial surgery (Schimming, 2004) however resorbable polymers degrade when expose to body fluid and therefore show poor mechanical properties for load-bearing orthopedic applications. Non-resorbable additives such as polyamide fibers can significantly enhance polymer properties (Mehboob and Chang, 2014) but the need for a second surgery to remove them lead to the use of completely resorbable and/or bioceramics as reinforcement for the composites. Resorbable polymeric composites can be generated by combining HA/PLA, TCP/PLGA, and phosphate glass fiber/PLA whereas their degradation rates can be controlled by the addition of fibers, coatings and various coupling agents (Parsons et al., 2009; Haque et al., 2010; Ahmed et al., 2011; Harper et al., 2012). Moreover, Fielding et al. found increased average density, faster cell proliferation, and a 2.5 fold increase in compressive strength of tricalcium phosphate by incorporating silica (SiO(2)) (0.5 wt%) and zinc oxide (ZnO) (0.25 wt%) dopants into the biomaterial (Fielding et al., 2012). Other study have also found increased compressive strength of almost 2 MPa in a zirconia (ZrO2)/ β-tricalcium phosphate (β-TCP) composites. Interestingly, they noted a suitable environment for osteoblast survival and bone regeneration *in vitro* when the composite was used (Alizadeh et al., 2016).

Other studies using CaP biomaterials modified with PEGylated poly (glycerol sebacate) (PEGS) polymers also demonstrate optimization of their mechanical characteristics and bioactivity in BTE applications (Ma et al., 2016). Repair of bone defects can be effective when implementing chitosan-β-tricalcium phosphate composite, as a study by Yang et al. demonstrated effective osteogenesis and vascularization after MSCs injection (Yang et al., 2015). The ratio of composite biomaterials also plays a role in its effectiveness as was demonstrated in a study comparing porous composite biomaterials of PGA/beta-TCP, in a 1:1 and 1:3 weight ratio, for repair of critical defects in rat femoral medial-epicondyles. They noted that by 90 days, bone replacement was almost complete and appeared healthy. Bone mineral density and biodegradation of the repair was highest amongst the PGA/beta-TCP groups, with the 1:3 biomaterial ratio showcasing the highest results (Cao and Kuboyama, 2010).

While different ratios and combinations of composites have been investigated, other studies have examined techniques to improve functional performance of poly(ε-caprolactone)/tri-calcium phosphate (PCL/TCP) biomaterials by coating them with carbonated hydroxyapatite (CHA)-gelatin composite via biomimetic co-precipitation. They found an increased proliferation rate of cultured porcine BMSCs of about 2.3 times that of non-coated CHA-coated composites. In addition, the CHA-gelatin composite coated PCL/TCP biomaterials stimulated BMSCs osteogenic differentiation the most (Arafat et al., 2011). Overall, results of composite biomaterials show promising results as frameworks for BTE.

### Growth factors

Physiologically growth factors are usually stored in bone ECM, actively released after injury and play crucial role in bone repair with bone morphogenetic proteins (BMPs), vascular endothelial growth factor (VEGF), fibroblast growth factor (FGF), platelet derived growth factor (PDGF), transforming growth factor-β1 (TGF-β1), and insulin-like growth factor 1 (IGF-1) being the major regulators of bone remodeling cascade. The therapeutic use of recombinant growth factors is based on the hypothesis that through appropriate signaling they induce and/or accelerate the bone healing process. However, only a number of recombinant growth factors (such as BMP-2 and BMP-7) have achieved commercial success due to limitations related to their safety, cost-effectiveness, low stability, short half-life and rapid deactivation of their actions in *in vivo* settings. To optimize recombinant growth factor delivery *in vivo* three different strategies can be used physical immobilization, non-selective covalent immobilization through growth factor's functional residues, and bioaffinity immobilization. Lately, biocompatible nanoparticles combined with protein immobilization strategies have been shown to augment the delivery and effectiveness of growth factors, and to mimic the physiological bone healing cascade (Wang et al., 2017).

#### BMP

Discovered more than 50 years ago as an inducting agent for *de novo* bone formation (Urist, 1965), bone morphogenetic proteins (BMPs) are today recognized as the most efficient growth factor family in aiding the healing of large bone defects. Two of its members constitute the only osteoinductive growth factors approved by the FDA for clinical use, commercialized as INFUSE (rhBMP-2, Medtronic) and OP-1 Putty (rhBMP-7/OP-1, Stryker) (Cahill et al., 2015). To date, over 20 BMPs have been identified, and several have been shown to play important roles in the induction of osteogenesis, including BMP-2, 4, 5, 6, and 7 (Ferreira et al., 2013; Fischerauer et al., 2013). Clinically, BMPs have been utilized to heal open tibial fractures and nonunions (Kanakaris et al., 2008), form new bone in the disc spaces for spinal fusion procedures (Brandoff et al., 2008), and induce the formation of new bone in dental procedures (Lan et al., 2007). However, only BMP-2 has been shown to be absolutely essential to the osteogenic process, while BMP-2 and BMP-7 are approved for clinical use in the healing of major bone defects (Chen et al., 2012).

BMP-7, also termed osteogenic protein-1 (OP-1), has been identified as an osteogenic factor in the repair of critical sized long bone defects and craniofacial bones (White et al., 2007). Early studies have shown that treatment with BMP-7-infused collagen sponges are equally efficacious in healing fracture nonunions as autografts and demineralized bone matrix (Geesink et al., 1999). A randomized controlled trial of 120 patients with long bone nonunions treated with BMP-7/collagen constructs demonstrated a significant increase in union rate (86.7%) compared with an alternative therapy (i.e., PRP−68.3%) (Calori et al., 2008). In parallel, there have been numerous long-term observational studies demonstrating the safety and efficacy of rhBMP-7 in the treatment of long bone nonunions (Kanakaris et al., 2008).

Murine models have shown that BMP-2 is not an essential part of prenatal limb formation, however, it is absolutely necessary during postnatal fracture healing. Successful bone engraftment depends upon BMP-2 as a stimulus for the repair process, and a loss in BMP-2 in the host or donor periosteal cells results in a failure of callus formation or subsequent bone healing (Wang et al., 2011). A 2002 randomized control study looking at 450 patients with open tibial fracture demonstrated that patients treated with collagen sponges loaded with rhBMP-2 had significantly better fracture healing post-operatively compared to control patients (Govender et al., 2002). A large body of literature exists documenting the successful use of BMP-2 in osteoinduction and bone healing, but there still remains concern regarding the large dose of BMP required for treatment, modes of delivery, heterotopic ossification, and the possibility of an increased risk of cancer (Cahill et al., 2015). In addition to these concerns, major complications have been related to the “off-labeled” use of INFUSE (BMP-2) in spine surgery, most notably being marked dysphagia, seromas and hematomas, swelling, and/or the need for intubation/tracheostomy (Epstein, 2013).

#### VEGF and FGF

Bone is a highly vascularized tissue that requires oxygen, minerals, growth factors, and other signaling factors to survive. When a fracture disrupts the blood supply, growth factors are required to re-vascularize the damaged area to bring osteoprogenitor pericytes and promote the formation of new bone (Di Bella et al., 2008). Vascular endothelial growth factor (VEGF) is a fundamental mediator of angiogenesis in fracture healing. The growth of the new blood vessels allows for the delivery of mesenchymal progenitors which differentiate into osteoblasts (Maes et al., 2010; Chen et al., 2012), a process that is further stimulated by the chemotactic effect of VEGF on MSCs (Mishima and Lotz, 2008). VEGF has also been found to induce differentiation of progenitor cells into osteoblasts through the secretion of osteotropic growth factors from endothelial cells directly induced by VEGF (Wang et al., 1997). Early studies have demonstrated that blockade of VEGF in mice and primates by monoclonal antibodies suppresses nearly all blood vessel invasion in the area, consequently impairing trabecular bone formation (Ryan et al., 1999), while stopping anti-VEGF treatment results in the return of normal bone growth (Ferrara et al., 2003). Street *et al*. found that mice with neutralized VEGF receptors had decreased angiogenesis and bone formation in femoral fractures (impaired endochondral ossification), as well as significantly inhibited healing of tibial cortical bone defects (compromised intramembranous ossification), demonstrating how VEGF is essential to bone healing through chondrocyte intermediate and direct repair mechanisms (Street et al., 2002). Additionally, administration of VEGF through PLGA biomaterial has been shown to significantly increase neovascularization and bone regeneration in irradiated osseous defects in rats. Taken together, these studies further demonstrate the necessary role VEGF plays in the fracture healing cascade.

Fibroblast growth factor, specifically FGF-2, is another signaling peptide implicated in angiogenesis and bone regeneration. FGF-2 administered via collagen sponge to rat calvarial critical-size bone defects has been shown to promote greater osteoblast differentiation as well as higher blood vessel and bone volume generation in a concentration-dependent manner (Kigami et al., 2013). Additionally, there has been some suggestion that FGF could play a role in up regulating VEGF, helping to augment the bone healing process through cross-talk with other growth factors (Rabie and Lu, 2004).

#### PDGF

Platelet derived growth factor (PDGF) is a signaling molecule that plays a critical role in angiogenesis and the migration and proliferation of MSCs. Though there are multiple isoforms, PDGF-BB is considered the universal growth factor in this family due to its ability to bind to all isoforms of the PDGF receptor (Hollinger et al., 2008). PDGF is a chemoattractant for pericytes, facilitating their recruitment and attachment to vascular endothelial cells to aid in the structural stability of newly forming vasculature (Caplan and Correa, 2011a). It has been suggested that PDGF, secreted by endothelial cells, facilitates the release of pericytes from the vessel wall. These free pericytes then give rise to MSCs, which can be stimulated by PDGF to differentiate into osteoblastic progenitors, facilitating fracture repair. It should be noted that the release of the pericytes/MSCs from the vasculature only happens during active angiogenesis, such as what occurs during new bone formation (Caplan and Correa, 2011a). When a fracture occurs, an inflammatory response releases large numbers of bioactive factors in the site of injury, including a high concentration of PDGF-BB from aggregating platelets potentially facilitating the release of pericytes in the fracture site, providing progenitors for the formation of osteoblasts that will lay down new bone.

Due to its mechanism of action, exogenously applied PDGF only remains at the fracture site for a few days taking several weeks to show effects of increased bone growth and volume. Animal studies examining the effects of PDGF-BB on tibial fractures have found that PDGF-BB delivered with a collagen matrix significantly enhances fracture repair compared to controls (Nash et al., 1994). A large, prospective, randomized controlled study involving 11 clinical centers compared the effectiveness of rhPDGF-BB to beta-tricalcium phosphate (beta-TCP) and found PDGF-BB to be safe and more effective in treating periodontal osseous defects compared to beta-TCP (Nevins et al., 2005). Given the important role PDGF plays in the induction of osteoblastic activity, vascular growth, and subsequent fracture healing, there is enormous potential in utilizing PDGF in clinical orthopedic settings.

#### IGF-1 and TGF-β1

The skeleton undergoes constant remodeling, a process that involves close coupling of bone resorption and formation through osteoclasts and osteoblasts, respectively (Raggatt and Partridge, 2010). During osteoclastic activity, signaling factors are released from the bone matrix through the resorption process which helps recruit osteoblastic BMSCs to the site, inducing bone formation. It has been largely demonstrated that transforming growth factor-β1 (TGF-β1) and insulin-like growth factor 1 (IGF-1) each play critical roles in the coupled pathway of skeletal remodeling.

TGF-β1 is one of the most abundant cytokines present in the bone matrix, residing in its inactive form by remaining non-covalently bound to a latency-associated protein (LAP) until cleaved and activated by osteoclasts. Once active, TGF-β1 functions as a chemotactic agent to recruit local MSCs for osteogenic differentiation through the SMAD intracellular signaling transduction pathway. A study by Tang et al. used mice to demonstrate that TGF- β1 is necessary and responsible for inducing migration of BMSCs to skeletal remodeling sites in response to osteoclastic bone resorption. This study also showed that an induced mutation in the *TGF-*β*1* gene leading to overexpression of the active form of TGF-β1 resulted in defective bone remodeling due to formation of osteoblastic cell clusters and impaired migration of BMSCs to bone resorption sites (Tang et al., 2009). These results are consistent with human skeletal disorders that result from mutations causing premature activation of the TGF-β1 gene (Janssens et al., 2000). Tang et al. was able to partially rescue the uncoupled bone resorption in TGF-β1 mutant mice through a receptor inhibitor, demonstrating a possible mode of therapy in treating bone remodeling diseases (Tang et al., 2009).

Even though TGF-β1 is responsible for recruiting BMSCs to the site of bone resorption, they do not provide the signal for active osteoblastic differentiation. Instead, IGF-1, which is also one of the most abundant growth factors present in the bone matrix (Seck et al., 1998), is responsible for creating the osteogenic microenvironment required for differentiating recruited BMSCs into osteoblasts (Xian et al., 2012). In a study by Xian et al., IGF-1 knockout mice (*Igf1r*^−/−^) showed a reduction in mature osteoblasts at the site of bone remodeling as well as a reduction in bone formation, demonstrating how IGF-1 signaling is necessary for bone remodeling. They were able to determine that when IGF-1 is released from the bone matrix during osteoclast activity, it is able to activate mTOR signaling to stimulate osteoblastic differentiation of BMSCs recruited by TGF- β1. Additionally, IGF-1 levels in the bone matrix were found to be low in rats with age-related reduction of bone mass (Xian et al., 2012). This is consistent with previous knowledge that diseases involving low IGF-1 serum and matrix concentrations have high incidence of low bone mass and high risk of osteoporosis and fracture (Langlois et al., 1998). Delivery of exogenous IGF-1 to aged rats was enough to stimulate some new bone formation (Xian et al., 2012). Since IGF-1 is necessary for the maintenance of healthy bone mass, it is worth further investigating its therapeutic potential in preventing disease states that put patients at high risk for fracture.

### Mechanical stimulation

As clinical demand for bone grafts to treat congenital and trauma related skeletal defects continues to increase, the method of seeding hMSCs onto biological and synthetic biomaterials along with osteoinductive growth factors has been a significant advancement in the field of tissue engineering. However, the size of the tissue constructs that can be created under static conditions is greatly limited due to diffusional constraints of nutrients reaching bone cells which have very high metabolic requirements (Grayson et al., 2011). A solution to this problem is the utilization of perfusion bioreactors which can effectively disseminate nutrients and oxygen throughout graft constructs with a core larger than 200 μm (generally thought to be the upper limit for oxygen diffusion and a bone graft in static culture). In addition to convective transport of nutrients and waste, the dynamic flow of perfusion bioreactors creates a mechanical stimulus that enhances osteogenesis and mineral deposition of cells in the graft (Gomes et al., 2003). It has been shown in various studies that use of a bioreactor allows for the cultivation of functional, clinically-sized bone grafts that can be used for transplantation (Grayson et al., 2011).

*In vitro* studies have shown that marrow stromal osteoblasts significantly increase mineralization on 3D biomaterials with increasing flow rate in a perfusion bioreactor (Bancroft et al., 2002). Moreover, medium perfusion rates from 0.01 to 0.2 mL/min appear to be optimal for increasing the number of viable cells, as perfusion rates approaching 1 mL/min results in substantial cell death (Cartmell et al., 2003). The mechanical shear stress stimulation from the perfusion of the bone cells is a strong stimulant for osteogenic growth. In fact, there are several studies providing evidence that the shear stress from fluid flow could be a stronger stimulus for osteoblastic activity compared to hydrostatic compression or deformation of the extracellular matrix (Bancroft et al., 2002). Despite these advantages, one problem regarding bone grafts constructed in perfusion bioreactors is their inherent lack of vasculature, which can lead to a necrotic core once implanted. Ball et al. investigated the use of 3D printed vascular structures applied in conjunction with a perfusion bioreactor and found that cell viability increased by 50% within the core graft constructs (Ball et al., 2016). Though it remains to be seen if tissue-engineered bone grafts can replace the current gold standard autograft in fracture repair, perfusion bioreactors and the advances in spatial resolution provided by 3D printing, as well as growing understanding of stem cell differentiation into osteochondral structures (Stephenson et al., 2017) provide tools that can be used to increase the viability and osteogenic potential of bone grafts for therapeutic use. In addition, as noted by Forrestal et al. culturing and integrating unique geometry in patient-specific grafts by coupling bioreactors and biomaterial s of vary shapes, complexities, and sizes, remains an area of investigation that could yield positive results in tissue engineering (Forrestal et al., 2017).

## Cell-based therapy applications in bone defects

Several factors determine the success of cell-based therapies for bone defects, including (1) the type of cells and vehicle used to transport them, (2) final cell concentration, (3) immature or progenitor cell status, (4) time of therapy, (5) mode of administration and (6) availability of osteoblastic progenitors at the fracture site. We have discussed some of these factors above in relation with cells, biomaterials or growth factors employed. However, the availability of osteoblastic cells at the fracture site constitutes another critical factor, which in fact can be manipulated. Overall, recruitment (i.e., homing) of endogenous MSCs to bone defect sites presents benefits and limitations when compared to exogenously administered autologous or allogeneic MSCs. Different strategies to induce homing of endogenous MSCs to bone defects are reviewed in Herrmann et al. (2015). On the one hand, and as mentioned before, homing of endogenous MSCs to bone defects can be triggered by locally injected growth factors combined with various biomaterials. In such cases, endogenous MSC recruitment does not involve *ex vivo* MSC manipulation that putatively affect their phenotypic and molecular profiles, thus having easier regulatory pathways. On the other hand, in some cases the recruitment of endogenous MSCs might not be adequate as the MSC functionality has been shown to be affected by intrinsic factors such as patient's pathological conditions (for example type 1 diabetes and autoimmune conditions) and age. In addition, bone segmental defects where bone loss cannot be compensated even by autologous bone grafting there is need to combine all different aspects of BTE including stem cells, growth factors, scaffolds and mechanical stimulation for effective bone regeneration (Figure 1). For these reasons, and not to discriminate against such approaches, we will only present a couple clinical scenarios using exogenously-administered cells, complemented by a list of available clinical studies using expanded MSC to treat bone defects (Table 2).

**Table 2:** Clinical studies using cultured MSCs for the treatment of bone defects.

**References**	**Cell type**	**Biomaterials/grafts**	**Delivery method**	**Patient's group**	**Average follow up**	**Outcome**
Kawate et al. (2006)	BM MSCs	β-TCP ceramics and free vascularized fibula	Local implantation of BM MSC/ β-TCP composites with free vascularized fibula	Steroid-induced osteonecrosis *N* = 3	27–48 months	Osteonecrosis did not progress any further and early bone regeneration was observed
Quarto et al. (2001); Marcacci et al. (2007)	BM MSCs	Porous HA ceramic	Local implantation, 2.0 × 10^7^MSCs per ml mixed with biomaterial	Large long bone defects *N* = 3, 4–7 cm segment from tibia, ulna, humerus	6–7 years	No complications observed. Complete fusion between implant and host bone 5–7 months post surgery. At 6–7 years post surgery good integration was maintained and no late fractures observed
Kim et al. (2009)	Osteogenically differentiated BM MSCs	–	Local injection, 1.2 × 10^7^ MSCs per 0.4 ml mixed with fibrin at 1:1 ratio	Various long bone fractures *N* = 64	2 months	No complications observed. Autologous osteoblast injection resulted in significant fracture healing acceleration
Zhao et al. (2012)	BM MSCs	–	Local injection, 2.0 × 10^6^ MSCs in 2 ml of saline	AVN of femoral head *N* = 53	5 years	No complications observed. At 5 years post surgery only 2 of the 53 BM MSC-treated femoral heads progressed and underwent vascularized bone grafting. Improved measures of femoral head function and decreased volume of the necrotic lesion
Giannotti et al. (2013)	Osteogenically differentiated BM MSCs	–	Local implantation, 0.5–2.0 × 10^6^ MSCs in 2 ml of fibrin clot	Upper limb non-unions *N* = 8	6 years and 3 months	No complications observed. All patients recovered limb function with no evidence of tissue overgrowth or tumor formation
Aoyama et al. (2014)	BM MSCs	β-TCP ceramics combined with vascularized bone grafts	Local implantation, 0.5–1.0 × 10^8^ MSCs mixed with β-TCP and vascularized bone grafts	AVN of femoral head *N* = 10	24 months	No complications observed. All procedures were successfully performed and some young patients with extensive necrotic lesions with pain demonstrated good bone regeneration with amelioration of symptoms.
Cai et al. (2014)	BMMNCs and UC MSCs	-	Infusion in femoral artery of 60–80 mL of BMMNCs and 30–50 mL of UC MSCs	AVN of femoral head *N* = 30	12 months	No complications observed. After the treatment, 28/30, 26/30, and 26/30 of patients showed relief of hip pain, improvement of joint function, and extended walking distances, respectively.

### Delayed fracture healing

Nonunions are complications that may occur 6 months following incomplete healing of a bone fracture. Studies have compared the function and pools of BMSCs and circulating endothelial progenitor cells (EPCs) in atrophic nonunion patients to healthy subjects in an effort to better understand the molecular and cellular mechanisms which lead to nonunions. Patients with nonunions were shown to exhibit a decreased pool of BMSCs and changes in the serum levels of chemokines and growth factors required for their recruitment and proliferation (Mathieu et al., 2013). Although the literature regarding stem cell use in the treatment of nonunions is limited to pre-clinical animal models, there have been reports of successful use of autologous BMSCs combined with calcium sulfate (CaSO4) to clinically and radiologically heal nonunions 2 months after implantation (Bajada et al., 2007). Another example involves the successful implantation of engineered osteogenic bone discs around the osteosynthesis material (i.e., intra-medullary nail in this case), in a patient with a critical bone defect of 72 mm in the distal tibia. The engineered bone discs were made with decellularized bovine bone matrix (DBM) seeded with autologous bone marrow aspirate and cultured *in vitro* before implantation. In this typical tissue engineering approach, the engineered implant demonstrated good integration with host tissue and functional clinical outcomes as early as 6 weeks post-surgery (Hesse et al., 2010).

Pre-clinical studies have used rat models to evaluate the efficacy of local injections and cell sheets of BMSCs to treat nonunions. BMSCs have shown the ability to complete bone bridging with woven bone within rat femoral osteotomies when compared to their controls (Shimizu et al., 2015). Using cell sheet transplantation of BMSCs cultured using dexamethasone and ascorbic acid phosphate has also been shown to aid in the repair of nonunions. Researchers demonstrated that transplanted cells without the use of biomaterials has the ability to differentiate into an osteogenic lineage and could contribute to hard tissue reconstructions in cases involving nonunions (Nakamura et al., 2010). Studies have also proven that BMSCs injected into bone defects have the potential to not only remodel bone, but to provide a greater degree of biomechanical stiffness when compared to contralateral uninjured limbs. For instance, in a study by Kallai et al., the biomechanical and microarchitecture properties of repaired murine radius bones following implantation of MSCs was found to have an axial stiffness 2 times higher compared to contralateral limbs when repaired with MSCs (Kallai et al., 2010).

Bone marrow aspirate concentrate (BMAC) has been explored as a viable source of BMSCs for the treatment of bone nonunions. BMAC is a heterogeneous cell mixture containing various cell types including hematopoietic and mesenchymal progenitors (i.e., BMSCs) along with EPC, monocytes, granulocytes and other cell types. Several studies have been reported using BMAC for nonunions, bone defects, distraction osteogenesis and others for potential complications of the therapy. The overall consensus of those studies is the clinical efficacy of BMAC inducing bone healing, its superiority to bone grafts, and reduced complications (Fayaz et al., 2011; Imam et al., 2017).

### Avascular necrosis of the femoral head

Avascular necrosis of the femoral head (AVN) is a disease which affects the younger population resulting in several complications. AVN can be idiopathic or secondary to chemotherapy, steroid therapy, trauma, chronic alcohol use, or sickle cell disease. Old reports have shown that more than 80% of patients with AVN develop end-stage osteoarthritis necessitating joint replacement (Mont et al., 1996). As a result, other modalities have been investigated in an effort to mitigate the progression of joint disease, specifically therapies using core decompression, cell-based therapies, or a combination of the two.

Core decompression (CD) provides relief by decreasing the intra-medullary pressure, which alleviates the symptoms but does not impact progression of the disease (Radke et al., 2003). It is typically reserved for patients in the early stages of osteonecrosis, prior to mechanical failure/collapse. Compared to conservative-treatment, CD can effectively relieve hip pain from AVN, however, there is no difference when compared to conservative treatment in preventing collapse of the femoral head from osteonecrosis as early established (Koo et al., 1995). Incomplete reconstruction of the femoral head secondary to deficiency of osteoprogenitor cells in the proximal femur may contribute to the failure of CD in preventing collapse of the femoral head.

In an effort to prevent the progression of AVN, studies have investigated the use of cells-based therapy in treating hip osteonecrosis. One highly explored method involves implanting BMAC into the osteonecrotic zone on the femoral head to create an environment of osteoblast differentiation and vascular proliferation to promote repair (Zhao et al., 2012; Hernigou et al., 2015). Studies have also investigated the efficacy of culture-expanded BMSCs compared to CD, revealing that the presence of BMSCs significantly increased Harris Hip Scores and decreased the volume of necrotic lesions when compared to CD (Hernigou et al., 2016). They concluded that autologous BMSCs, obtained from the iliac crest, can reliably provide a greater number of cells for efficient expansion and further femoral head implantation and could effectively delay or avoid its collapse. Finally, a meta-analysis comparing the clinical efficacy of core decompression with BMSC revealed that the cells-based group progressed to a significantly fewer number of grafting events as well as a significantly better clinical outcome compared to CD (Li et al., 2014).

Studies have also investigated how CD in conjunction with cell-based therapy can be beneficial for patients with AVN. Villa et al. revealed patients treated with a combination of BMSCs with CD, compared to controls and CD alone, had a lower risk of femoral head collapse (Villa et al., 2016). Other studies have replicated these results as Gangji et al. compared patients with AVN treated with CD or CD with bone marrow cells in the form of BMAC. They found that the group with cells had less pain and joint symptoms, as well as reduced incidence of fractural stages. These results were maintained after 60 months as there was a significant difference in failure rates between the two groups (Gangji et al., 2011). These results are supported by additional studies showing cells injected in conjunction with CD to provide a significant difference in the time to collapse when compared to patients treated with just CD (Gangji et al., 2004).

## Conclusions

A major challenge for the upcoming decade in the bone regeneration field is to elucidate the underlying cellular and molecular mechanisms of stem cell actions in bone defect therapeutics. In previous years, both pre-clinical and some clinical studies have shown beneficial effects, mainly with MSCs and osteoblastic progenitors in bone healing but the exact mechanisms of actions remain unclear. Although encouraging clinical results have been obtained by transplanting either uncultured MSCs or expanded MSCs, the exact dosage and route of application remain to be optimized and the fate of transplanted cells and their mechanisms of action need to be better monitored in larger clinical trials. On this basis, clinical applications of stem cell-based bone therapies are still limited due to various hurdles such as (a) ethical concerns related to ESCs and iPSCs therapeutic application, (b) proper phenotypic and molecular qualitative evaluation of stem cell products (c) laborious and expensive *ex vivo* expansion of adequate stem cell populations for effective *in vivo* applications c) putative immunological rejection of allogeneic stem cell infusion *in vivo* and donor-related differences and (d) other translational difficulties. Specifically, for the treatment of delayed fracture healing and avascular necrosis bone defects, local administration of stem cell therapies in combination with specialized osteo-conductive biomaterials, osteo-inductive growth factors and adequate mechanical stimulation may further resolve more effectively and more rapidly these complicated orthopedic conditions. Collectively, a better understanding of the functional roles of stem cells in health, aging and disease will provide the foundations for the design of novel and advanced therapeutic strategies for skeletal disorders.

## Author contributions

All authors contributed substantially to the conception or design of the work, the drafting of the work or revising it, reviewing for final approval, and agreement to be accountable for all aspects of the work in ensuring that questions related to the accuracy or integrity of any part of the work are appropriately investigated and resolved.

### Conflict of interest statement

The authors declare that the research was conducted in the absence of any commercial or financial relationships that could be construed as a potential conflict of interest.
